# The Biological Effect of Extremely Low Frequency Electromagnetic Fields and Vibrations on Barley Seed Hydration and Germination

**DOI:** 10.1100/tsw.2004.179

**Published:** 2004-10-20

**Authors:** Armine Amyan, Sinerik Ayrapetyan

**Affiliations:** UNESCO Chair-Life Sciences International Postgraduate Educational Center, 31 Acharyan st., Yerevan, 375040, Armenia

**Keywords:** water structure, water binding, seed swelling, root formation, germination

## Abstract

The changes of wet and dry weights and germination of barley seed in different periods of its swelling in nontreated (control), extremely low frequency electromagnetic fields (ELF EMF) )—treated, and extremely low frequency vibrations (ELFV)—treated cold (4°C) and warm (20°C) distilled water (DW) were studied. The metabolic-dependent seed hydration, dry weight dissolving, germination, and water binding in seed were modulated by preliminary EMF- and ELFV-treated DW. Frequency “windows” for the effect of EMF and ELFV on seed hydration, solubility, water binding in seed, and germination were discovered. These “windows” were different for EMF and ELFV, as well as in various phases of seed swelling. It is suggested that EMF-induced water structure modification has a different biological effect on the process of seed hydration, solubility, water binding in seed, and germination compared to ELFV.

## INTRODUCTION

At present, the biological effect of extremely low frequency electromagnetic fields (ELF EMF) can be considered as a proven fact, however, the cellular and molecular mechanisms through which this effect is realized are not yet fully understood. One of the most popular hypotheses is the "aqua" hypothesis, according to which the cell bathing aqua medium could serve as the main target through which the biological effect of EMF on cells and organisms is realized[1,2,3,4,5].

It has been shown that static magnetic field (SMF), ELF EMF, and extremely low frequency vibrations (ELFV) have a depressing effect on distilled water (DW) specific electrical conductivity (SEC)[4,6]. It was established that the inhibitory effect of those factors on SEC depended on their intensity and, in the case of LF EMF and ELFV, also on their frequencies[6]. The previous studies have shown the similarity of frequency "windows" for LF EMF and ELFV effects on water SEC, however, the question of whether or not these factors-induced water SEC depression have the same biological meaning for the cell, is still unanswered.

Previously it was shown that the pretreatment of aqua medium by the above-mentioned factors led to the modulation of the functional activity of microorganisms[6,7], sperm[8], contractile activity of the perfused snail heart[9,10], and neurons[11]. However, the comparative study of the frequency dependence of the biological effect of LF EMF and ELFV on different cells and organisms was not yet properly investigated. We suggest that such study would allow us to come to a close understanding of the physicochemical mechanisms through which the biological effect of both is realized. It is suggested that plant seed hydration and germination could serve as a more convenient experimental model for such study.

In barley seed, the growth of the vegetative organs closely correlates with the passing through of the stages of organogenesis. As do other germinating plants, barley goes through 12 stages of organogenesis. According to "Stron theory", the first stage of organogenesis of germinating plants begins with pre-embryo formation and ends with seed germination and the appearance of shoots. At room temperature in DW, the stage of organogenesis has lasted 72 h[12]. Usually, 4 stages of seed growth can be distinguished at room temperature: first stage, 2 h (water swelling until the critical moisture and active functioning of the ferments); second stage, 24 h (awakening); third stage, 48 h (germinal root formation); and fourth stage, 72 h (germination)[12].

Thus, on the basis of time-dependent study, seed wet weight and dry weight during incubation in control, EMF- and ELFV-treated cold or warm DW will be concluded on the effect of factor-induced water structure changes on the following processes:

1. Dissolubility of seed component
2. Water binding in seed
3. Passive, nonmetabolic dependent seed hydration
4. Metabolic-dependent seed hydration
5. Germination potential

Therefore, it is suggested that plant seed swelling in DW can serve as a very convenient experimental model for studying the biological effect of water structure changes on seed germination potential.

## METHODS

The seeds of spring barley (sort- Nutans 115, forming fibrous root systems, cleistogamous) were used. The seeds were cultivated in the Shirak Valley (Armenia) and were kindly supplied by the Echmiadzin Research Center of Agriculture and Plant Protection (Armenia). DW was obtained using the device DE-4-2M (Russian production, State Standard 64-1-721-91). DW has initial conductivity at room temperature (20ºC) in the range of 1–10 µs/cm. The latter depended on the "age" of DW, i.e., time passed after water distillation[6].

The water was gathered in tightly closed glassware and then poured out into two identical glass test tubes. The special setup was assembled (Institute of Radiophysics and Electronics [IRPhE] of Armenian NAS, Yerevan, Armenia) allowing the treatment of DW by EMF and ELFV. The block scheme of this setup is presented on Fig. 1. Glass test tube (1) with diameter 10 mm and volume 10 ml was used. The vibrator was controlled by the sine-wave generator (6) (GZ-118, Made in Russian Federation), the signal went to the double pole switch (8); in position I the generator functions as EMF and ELFV sources, while in position II, it functions as ELFV sources. To obtain ELFV waves. the vibrating device (3) was used, generating vertical vibrations by set frequency and intensity. The vibrator was constructed in the Department of Engineering at LSIEC on the basis of the IVCh-01 device (Russian production). To keep vibration intensity constant (30 dB) at different frequencies, a coil (4) with a feedback amplifier system (IRPhE, Yerevan, Armenia) was used. Thus, ELFV was transmitted to the test tube containing DW with

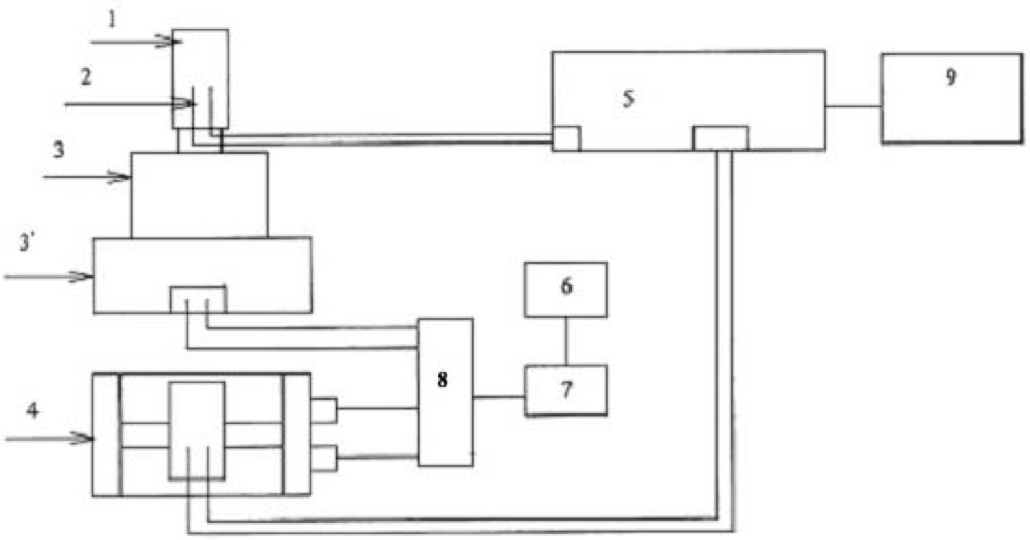

**FIGURE 1**. The setup for treatment of DW by LF EMF and ELFV. (1) Glass test tube with diameter 10 mm and volume 10 ml, (2) platinum electrodes, (3) mobile part of the vibrator, (3′) motionless part of the vibrator, (4) the coil, (5) the device for the measurement of DW SEC (conductometer), (6) generator of sinusoid vibration, (7) the low-noise amplifier, (8) the switch (has two positions: I is EMF and ELFV and II is EMF), (9) personal computer.

insignificant power dissipation. For concordance of high-impedance output of generator to low-impedance input of vibrator, a special power amplifier (IRPhE, Yerevan, Armenia) was used. ELFV frequency was controlled by a cymometer (CZ-47D, production of Russian Federation), while the intensity was measured by a measuring device (IRPhE, Yerevan, Armenia) that had a sensor on the vibration table. It was possible to keep the intensity of ELFV on a stable level at all frequencies, including resonance frequency (more than 200 Hz for the given setup).

EMF was generated by the controlled generator (6) and low-noise amplifier (7) on the coil (4) (IRPhE, Yerevan, Armenia). The coil had a cylindrical form 154 mm in diameter and 106 mm in height. The coil consisted of Helmholtz rings generating the homogeneous magnetic field. Rings of Helmholtz were formed by two equal ring coils located coaxially and parallel. The distance between ring coils was equal to their radius (77 mm). The magnetic field created by these rings had high homogeneity, for example, at a distance of 0.25 cm from the center of an axis strength differs from computed by formula only on 0.5 %

$$H = 71.6 \cdot \omega \cdot \frac{I}{R}.$$

DW was treated for 30 min by EMF and ELFV after which 10 ml of treated DW was added to each Petri dish (vol. ~45–50 ml), containing 20 seeds and this moment was considered as the starting time of seed incubation. To exclude the effect of light, the experiments were performed in the dark at 4 or 20°C.

Each experimental sample (version) consisted of 20 seeds. For statistical validity, each stage of the experiment was repeated 10 times. In each experiment, the initial weight of the seed was varied in the range of 40–50 mg. Before the experiments, all the seeds were weighed and this weight will be determined in the article as “wet weight$_0$”. After weighing, the experimental groups of seeds were incubated in nontreated and treated DW for 2, 24, 48, and 72 h. Thus, we had the possibility to calculate the quantity of the “free water” and dry substance in the seed in the different periods of its evolution, distinguished by the metabolic activity.

Before seed incubation in control and experimental medium, the wet and dry weights were determined separately for each seed. It was shown that the exudation of seed wet weight was 4.19 ± 0.004

mg and the level of seed hydration determined as 1 mg water/1 mg dry seed was 0.08 ± 0.004 mg for each seed. The following changes of wet and dry weights during seed incubation in control and experimental medium were expressed in percents compared with their initial value before the incubation.

The effect of factor-treated DW on time dependence of seed wet and dry weight changes was studied in different periods of incubation in cold and warm conditions. The value of seed dry weight was obtained by drying them at temperature 104ºC for 24 h in thermostat[14]. Seed hydration (gr.water/gr.dry weight) was counted as ([wet weight-dry weight]/dry weight). The reliability of results were $\alpha = 0.05$. In figures, where the range of variations is not observed, it means that their values are too small that are covered by symbols.

During the second stage of the experiments, the root formation and germination of barley seed were studied for 18 days (first 12 days in dark conditions and the following 6 days in light conditions) of incubation in nontreated (control), EMF- and ELFV-treated DW.

The length of the roots was calculated starting from the 8$^{th}$ day of incubation. The length of the germs was calculated starting from the 12$^{th}$ day of incubation. During the whole incubation period, the temperature regime was stable. Each Petri dish (vol. ~45–50 ml) contained 10 ml DW, which was renewed after each calculation of the length of roots and germs.

The length of roots and germs was measured in millimeters. All the data in this stage of the experiment were presented in percents, compared to the control, which was considered as 100%. The reliability of results were $\alpha = 0.05$.

The mean value was calculated based on the average, standard deviations, and confidence (Student-t test) with the help of the Excel computer program.

## RESULTS

It is obvious that in order to understand the biological mechanism of the effect of EMF- and ELFV-induced water structure changes on seeds, first it is necessary to study the passive water uptake by seeds when their metabolic activity is depressed (in cold condition) compared to the metabolic-dependent seed hydration in warm conduction.

As can be seen in Fig. 2A, the rate of seed hydration was gradually increased during incubation, which was accompanied by an increase of temperature sensitivity. The latter was more pronounced in the stage of seed germination during 48–72 h of incubation in warm DW.

The time-dependent changes of seed dry weight during 72-h incubation in DW (Fig. 2B) can be distinguished into three phases: (1) first 2 h, fast decrease; (2) 2–24 h, period of sharp increase; and (3) 24–72 h, period of increase at cold and decrease at room temperatures. It is obvious that the sharp decrease of dry weight during the first 2 h of seed incubation is the result of the exudation of their water-soluble components, while the "false increase" of dry weight after the end of 2 h of incubation can be explained by the increase of water binding in seed, i.e., to obtain a real value of dry weight it is necessary to dry the seed (at temperature 104$^{o}$C) longer than 24 h. However, the reason of active decrease of dry weight at 20$^{o}$C after 24 h of incubation can be interpreted as the exudation of seed dry weight or it can be the result of binding water decrease in seed. To come to the final conclusion on the latter, it is necessary to carry out more detailed investigations.

## LF EMF Effect

In Fig. 3, data are presented on time dependence of seed hydration in nontreated and in 4-, 10-, 15-, 20-, 50-Hz EMF-treated DW at cold (B) and room temperatures (A).

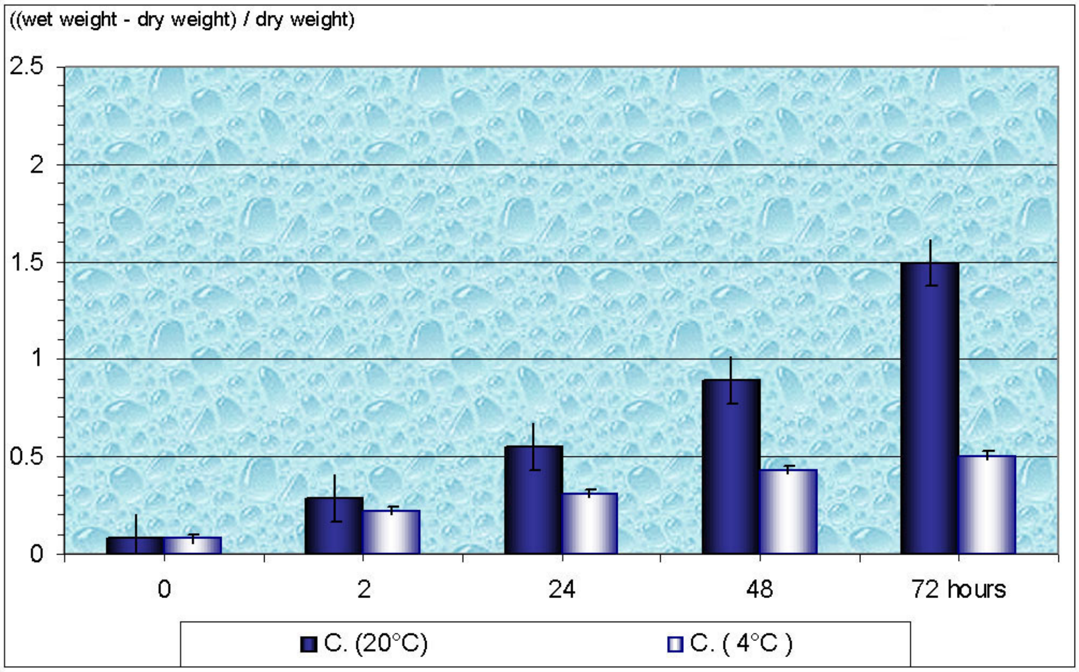

**FIGURE 2A.** The time-dependent seed hydration in nontreated DW in cold and warm conditions. On abscissa, the time (in hours) of seeds incubation; on ordinates, value of seed hydration (mg of $H_2O$ for 1 mg of dry weight) are presented.

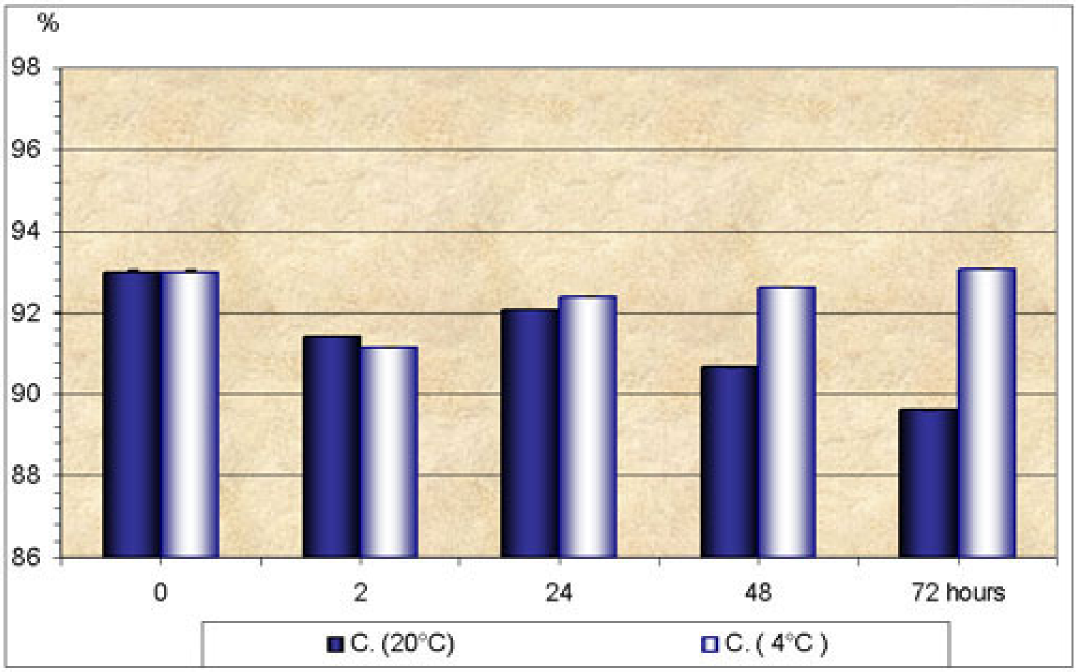

**FIGURE 2B.** The time-dependent changes of seed dry weight in nontreated DW in cold and warm conditions. On abscissa, the time (in hours) of seed incubation; on ordinates, percent of seed dry weight changes comparing to its wet weight$_0$.

*The confidence limits of computations were in range of 95%, reliability of results were 0.05%. On figures, where the range of variations is not observed, it means that their values are too small that are covered by symbols.*

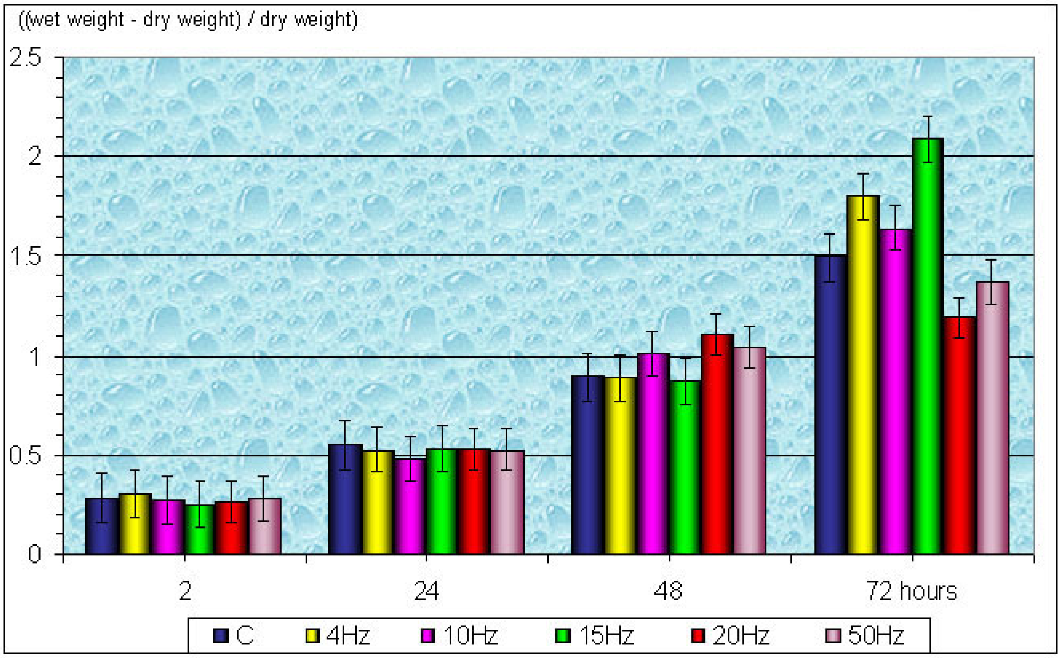

FIGURE 3A

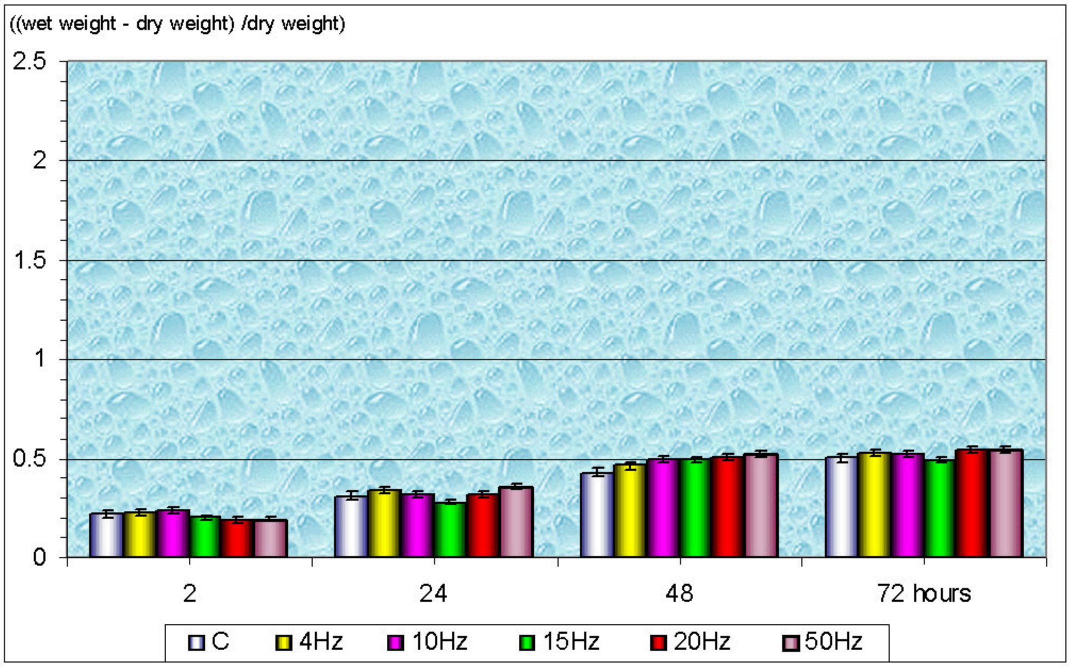

FIGURE 3B

**FIGURE 3.** The effect of nontreated (control) and LF EMF-pretreated DW on seed hydration during 72-h incubation. (A) Seed hydration at room temperature (20°C), (B) seed hydration in cold (4°C) DW. On abscissa, the time (in hours) of seed incubation; on ordinates, value of seed hydration (mg of $H_2O$ for 1 mg of dry weight).

As can be seen in Fig. 3, preliminary EMF-treated DW did not significantly modulate the kinetics of seed hydration in cold condition (B) and during the first 24-h incubation at room temperature (A), although, after the first 2-h incubation, the rate of hydration was comparatively higher than in the cold. The differences between seed hydration in warm and cold DW can be considered as a marker for their metabolic activity. The EMF sensitivity of seed hydration during the first 24-h incubation was not pronounced, while starting from the period of root formation (48-h incubation) at room temperature, it became more and more sensitive to water preliminarily treated by EMF. This sensitivity was frequency dependant and became more pronounced at the end of 72-h incubation at room temperature, i.e., during the most intensive period of seed germination[12]. The seed hydration at the end of 48-h incubation at room temperature in DW treated by 4- and 15-Hz EMF was approximately the same as in the control, while 10-, 20-, and 50-Hz EMF-treated DW had a clear activation effect on it (+13.1% ± 0.05, +25.2% ± 0.10, +16.6% ± 0.02, correspondingly). It is interesting to note that at the end of 72-h incubation, frequency “windows” for EMF effect on seed hydration were different than at the end of 48-h incubation. In 20- and 50-Hz EMF-treated DW, seed hydration was depressed compared to the control (–20.2% ± 0.22 and –8.2% ± 0.29, correspondingly, while 4-, 10-, and 15-Hz EMF had an activating effect on it (+20.7% ± 0.36, +9.8% ± 0.36, and +40.1% ± 0.35, correspondingly).

These data clearly show that the metabolic-dependent seed hydration is more sensitive to EMF-induced water structure changes than the passive one (in cold DW).

To estimate the effect of LF EMF-treated DW on solubility of seed components and water binding in seed, the time-dependent changes of seed dry weight during 72-h incubation in control and EMF-treated cold and warm DW were studied.

As can be seen in Fig. 4 (A,B), seed solubility and water binding in seed were significantly different in EMF-treated DW compared to the control. However, the curiously different frequency-dependant character of seed dry weight kinetics in DW treated by comparatively high (15, 20, and 50 Hz) and low (4 and 10 Hz) frequencies seems extremely interesting from the point of theoretical consideration and must be the subject for special detailed investigation. As can be seen on presented data, in the first 2 h in warm and cold conditions, 4-Hz EMF had no significant effect on rates of seed dry weight waste and 10 Hz has a slightly inactivating effect at room temperature, while 15-, 20-, and 50-Hz EMF in the same period had a more pronounced inactivation effect on this process, i.e., dry weight was increased, however, the highest effect was observed at 20 Hz (Fig. 4 A,B).

During the following period (24, 48, 72 h) of seed incubation in cold medium and at room temperature, the frequency-dependant effect on seed “dry weight” changes had complicated character; if in cold medium these changes could be explained by the modulation of the ability of seed to bind the water, at room temperature it could also be explained by different sensitivity of biochemical processes taking place in the seed, the number of which was increased during the period of germination, to the frequency-dependant changes of water structure.

The study of root formation in dark ($1^{st}$- to $12^{th}$-day incubation) and light ($12^{th}$- to $18^{th}$-day incubation) mediums (Fig. 5) and germination in light medium ($12^{th}$- to $18^{th}$-day incubation) (Fig. 6) have shown that 4-, 15-, 20-, and 50-Hz EMF have statistically significant inhibitory effects on both root formation and germination, while 10 Hz has a clearly activating effect on those processes. The latter effect was more pronounced at the end of the $8^{th}$-day incubation than during the following period incubation.

However, the frequency-dependant effect of EMF-pretreated DW on root formation and germination was significantly different from its effect on the kinetics of wet and dry weights of the seed.

As it was mentioned above, EMF-induced water structure changes can be realized by valence angle changes in water molecules as well as by mechanical vibration of its dipole molecules. Therefore, to estimate the contribution of vibration in the biological effect of EMF, in the next series of experiments the ELFV-treated DW effect on seed hydration, root formation, and germination was studied.

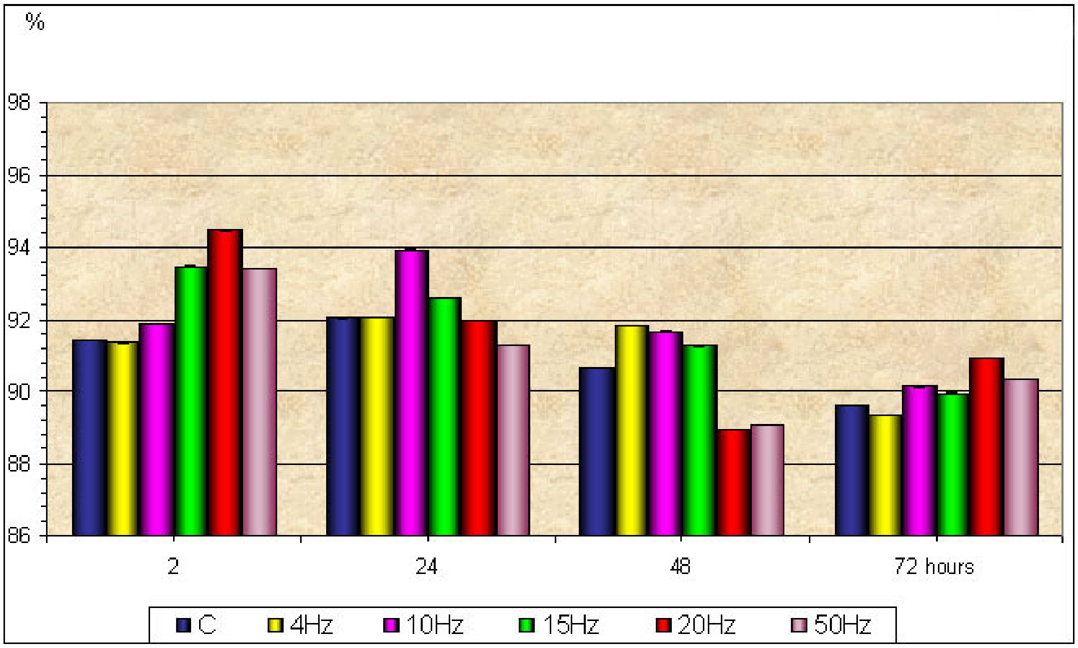

**FIGURE 4A**

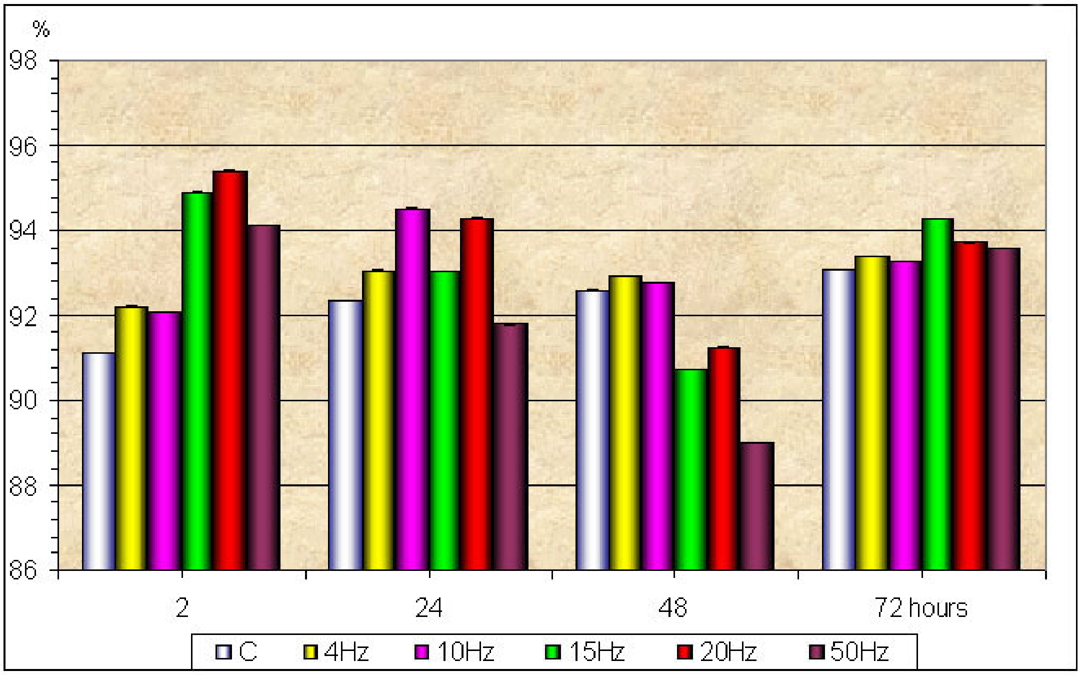

**FIGURE 4B**

**FIGURE 4.** The effect of nontreated (control) and LF EMF-pretreated DW on the changes of seed dry weight during 72-h incubation. (A) Seed dry weight at room temperature (20°C), (B) seed dry weight in cold (4°C) DW. On abscissa, the time (in hours) of seed incubation; on ordinates, percent of seed dry weight changes comparing to its wet $weight_0$.

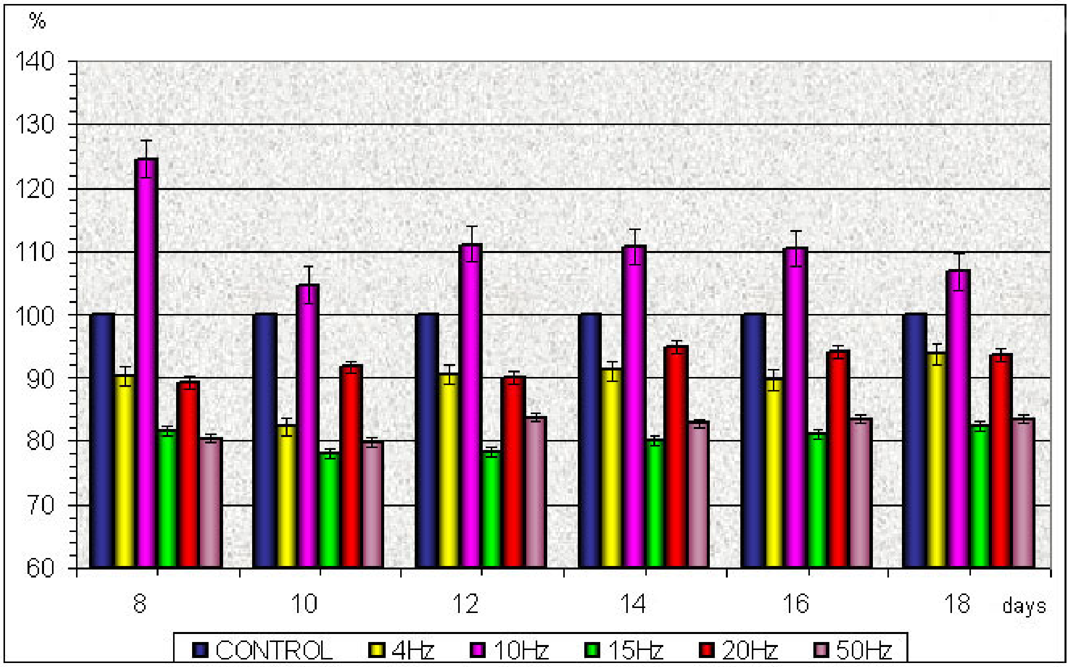

**FIGURE 5.** The time-dependent process of root formation during 18-days incubation in nontreated (control) and EMF-pretreated DW. On abscissa, the time (in days) of seed incubation; on ordinates, percent of the changes of root length comparing to the control.

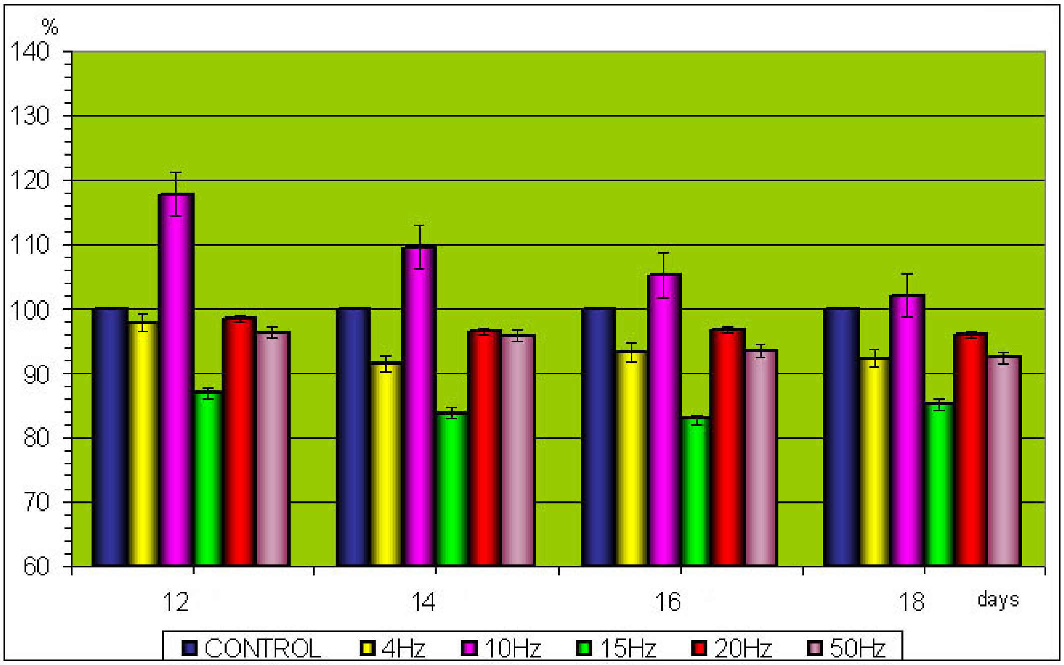

**FIGURE 6.** The time-dependent process of germination during 18-days incubation in nontreated (control) and EMF-pretreated DW. On abscissa, the time (in days) of seed incubation; on ordinates, percent of the changes of root length comparing to the control.

## ELFV Effects

The study of time-dependent dynamics of seed hydration and dry weight changes during their 72-h incubation in ELFV-treated cold and warm DW has shown that there are significant differences between kinetics of these parameters of seeds, incubated in control and ELFV-treated DW. As can be seen in Fig. 7, the effect of ELFV-treated DW has complicated character in the first period (2–48 h) of incubation at room temperature (Fig. 7A), while in the period of 48–72 h of incubation (period of germination), the clear increase of seed hydration rate in ELFV-treated DW was observed. The latter effect was ELFV frequency dependent; more pronounced effect was observed at frequencies of 4, 10, and 15 Hz. At 4-, 10-, 15-, 20-, and 50-Hz ELFV wet weight of seeds at the end 72-h incubation was increased in +26.9% ± 0.28, +34.3% ± 0.39, +28.5% ± 0.10, +12.5% ± 0.21, and +10.5% ± 0.35, correspondingly, compared to the control.

The study of dynamics of seed dry weight in ELFV-treated DW has demonstrated that during the first 2 h of seed incubation, 4- and 10-Hz ELFV had a slight depressing effect on the rate of dry weight decrease (the initial dry weight of seed was considered as 100%), while in the group of comparatively higher frequencies (15, 20, and 50Hz), this effect was more pronounced in the same period (Fig. 8). The sharp differences between kinetics of dry weight at 10 and 15 Hz of ELFV-treated DW allow us to suggest that there is a critical frequency “window” between them that could change the interaction between water molecules and seed components.

Thus, the values of dry weight after 2 h of swelling were 91.64% ± 0.005, 91.8% ± 0.006, 93.9% ± 0.006, 93.5% ± 0.006, and 94.4% ± 0.005 at frequencies 4, 10, 15, 20, and 50 Hz, correspondingly, while in the control it was 91.4% ± 0.013 (Fig. 8A).

After 72-h incubation in DW preliminarily treated by ELFV, the values of dry weight were 90.2% ± 0.011, 90.0% ± 0.009, 90.1% ± 0.010, 88.0% ± 0.011, and 91.1% ± 0.010 at frequencies 50, 20, 15, 10, and 4 Hz, correspondingly, while in the control it was 89.6% ± 0.007.

The differences between the rates of dry weight dissolving in control (as well as at 4- and 10-Hz ELFV-treated DW) and 15-, 20-, and 50-Hz-treated DW during the first 2 h of seeds incubation was less at room temperature than in cold DW (Fig. 8B). As in the case of DW treated by EMF, the character of the frequency-dependant effect of ELFV during the following periods of seed incubation was changed by the complicated manner.

The study of time-dependant root formation during 18-days incubation in nontreated and ELFV-treated DW have shown that this process was significantly changed in the DW preliminarily treated by ELFV, compared to the control (Fig. 9). It is interesting to note that at the end of the 8$^{th}$ day of incubation, 10- and 20-Hz ELFV-treated DW have activation (+5.66% ± 0.16 and +8.60% ± 0.17), while 4-, 15-, and 50-Hz have an inactivation effect on the process of root formation (–18.1% ± 0.26, –0.13% ± 0.27, and –19.17% ± 0.23, correspondingly). The similar frequency dependence was observed at the following light period of incubation (12$^{th}$–18$^{th}$ day of incubation). So, the effect of photosynthesis did not significantly change the character of the treated solution on the process of root formation.

The experimental data on the effect of ELFV-retreated DW on seed germination have also shown its frequency-dependent character: at the end of the 12$^{th}$ day of incubation in dark conditions, 10 and 20 Hz had a significant activation effect (+15.84% ± 0.28 and +7.14% ± 0.30), while 15 and 50 Hz had an inactivation effect, compared to the control (–9.44% ± 0.60 and –11.45% ± 0.56). 4-Hz–treated DW has a slight (+2.19% ± 0.66) activation effect on the process of seed germination (Fig. 10).

It is interesting to note that in the period of incubation in light conditions (12–18 days), when the process of photosynthesis was present, the inactivation effect of 15 and 50 Hz was potentiated (–14.33% ± 1.11 and –12.30% ± 1.03) and the slight activation effect of 4 Hz in dark medium was reversed and had a clearly inactivating effect (–10.51% ± 1.20). The activation effect of 10 and 20 Hz in dark conditions was slightly decreased in light conditions (+3.23% ± 0.55 and +1.37% ± 0.40). Thus, light conditions had an inhibiting effect on germination at all the investigated frequencies.

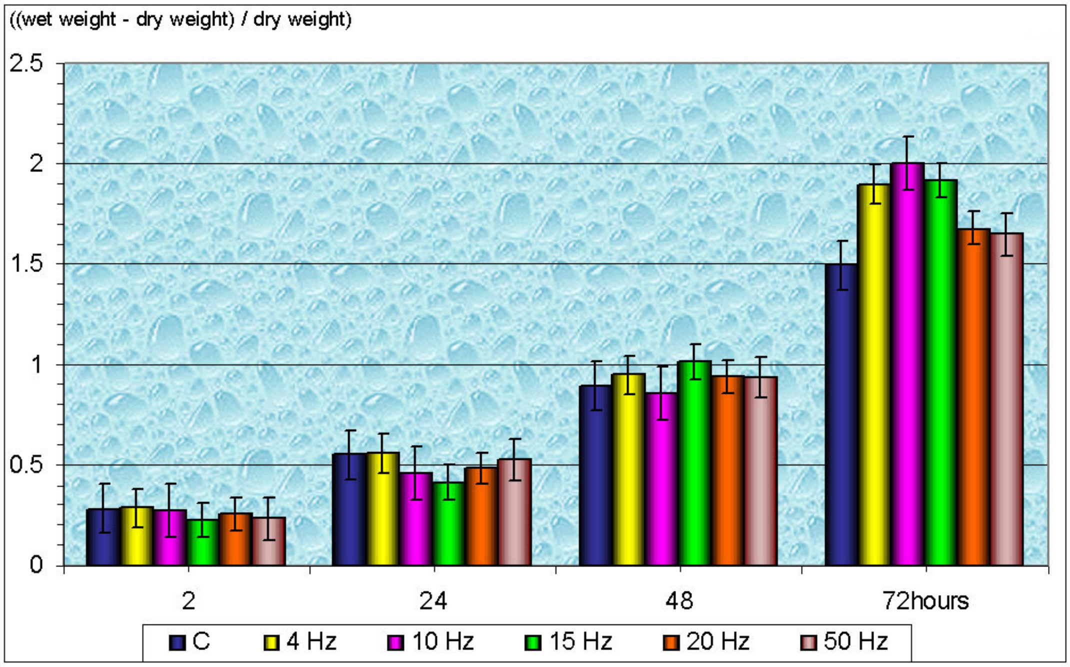

**FIGURE 7A**

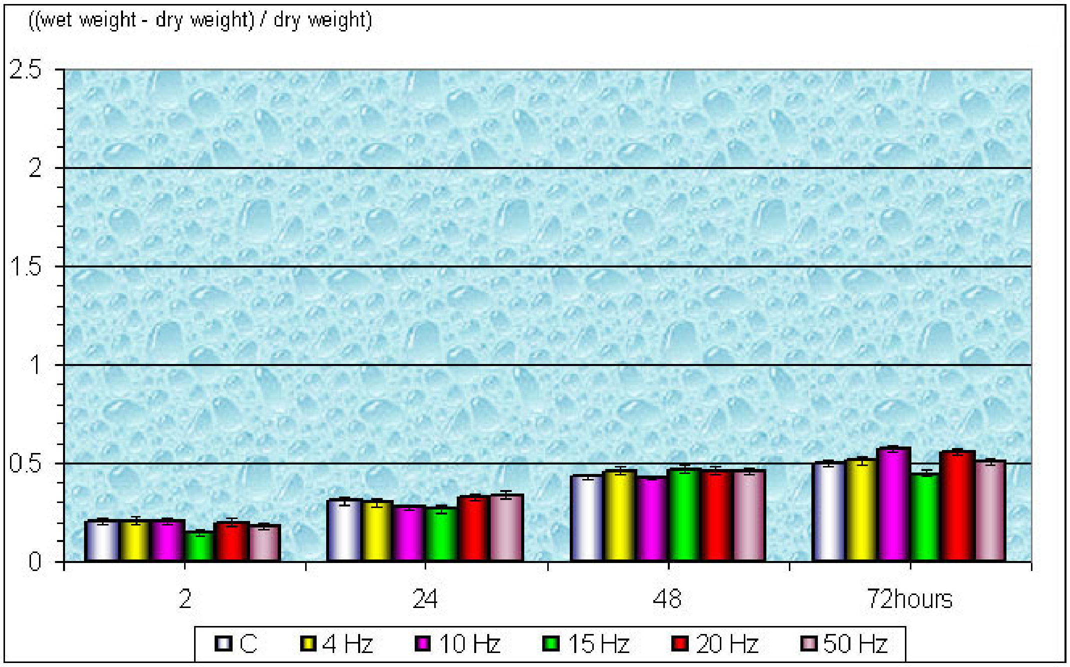

**FIGURE 7B**

**FIGURE 7.** The effect of nontreated (control) and ELFV-pretreated DW on seed hydration during 72-h incubation. (A) Seed hydration at room temperature (20°C), (B) seed hydration in cold (4°C) DW. On abscissa, the time (in hours) of seed incubation; on ordinates, value of seeds hydration (mg of $H_2O$ for 1 mg of dry weight).

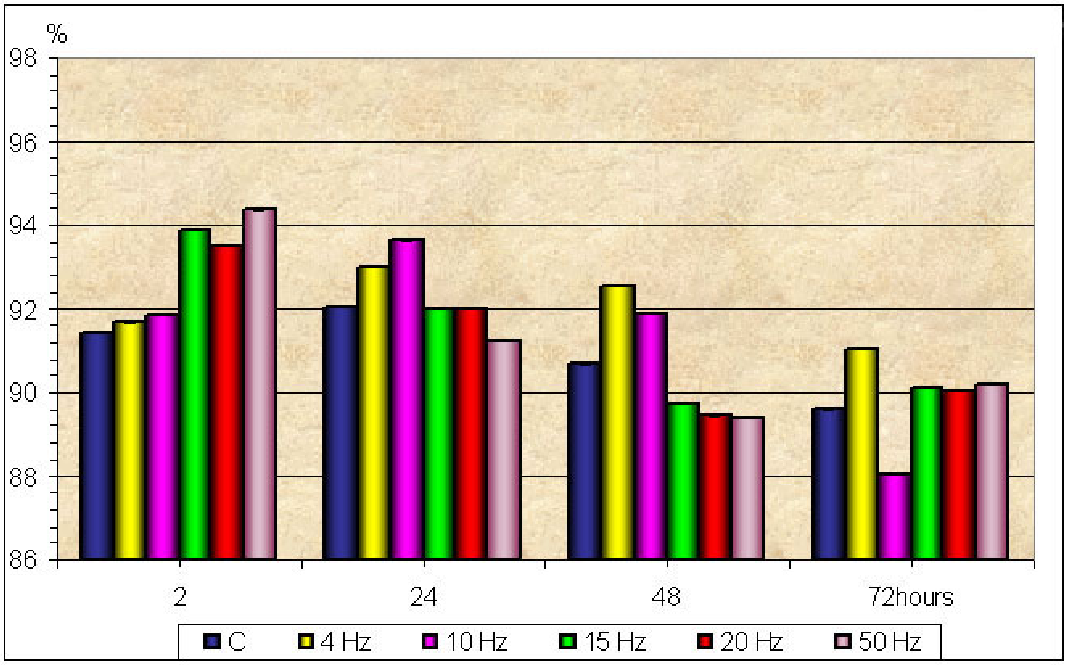

**FIGURE 8A**

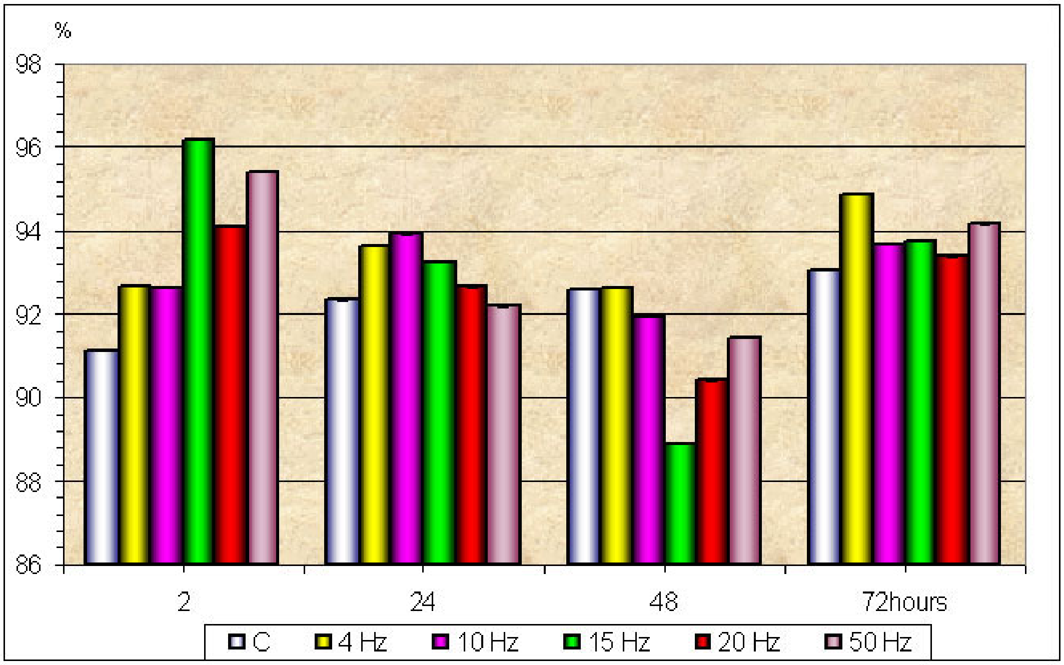

**FIGURE 8B**

**FIGURE 8.** The effect of nontreated (control) and ELFV-pretreated DW on the changes of seed dry weight during 72-h incubation. (A) Seed dry weight at room temperature (20°C), (B) seed dry weight in cold (4°C) DW. On abscissa, the time (in hours) of seed incubation; on ordinates, percent of seed dry weight changes compared to its wet weight$_0$.

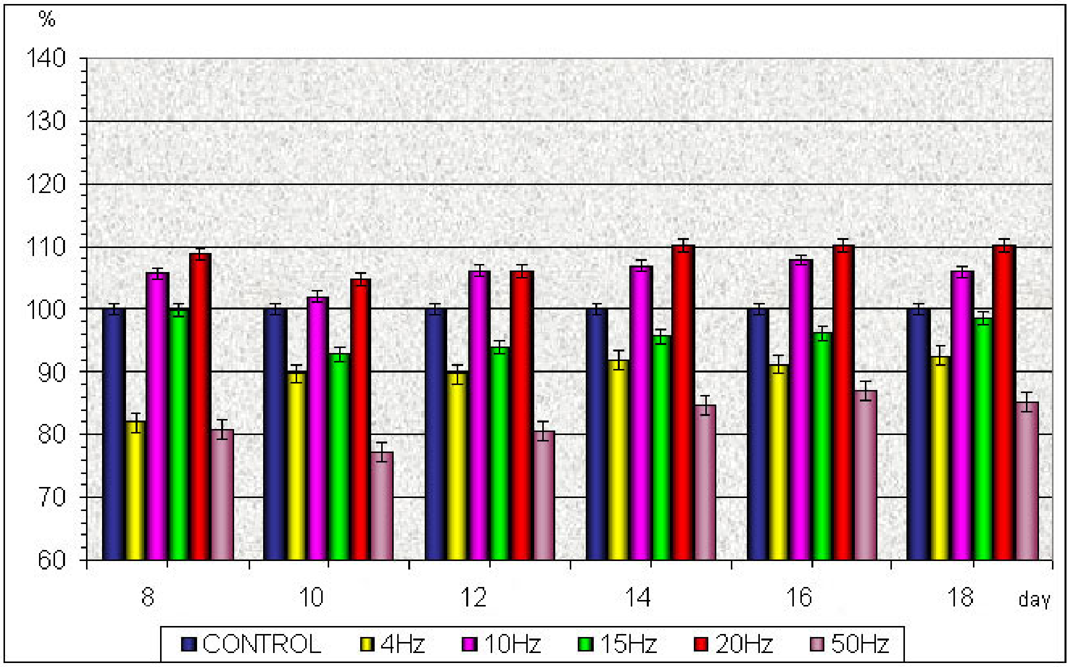

**FIGURE 9.** The time-dependent process of root formation during 18-days incubation in nontreated (control) and ELFV-pretreated DW. On abscissa, the time (in days) of seed incubation; on ordinates, percent of the changes of root length compared to the control.

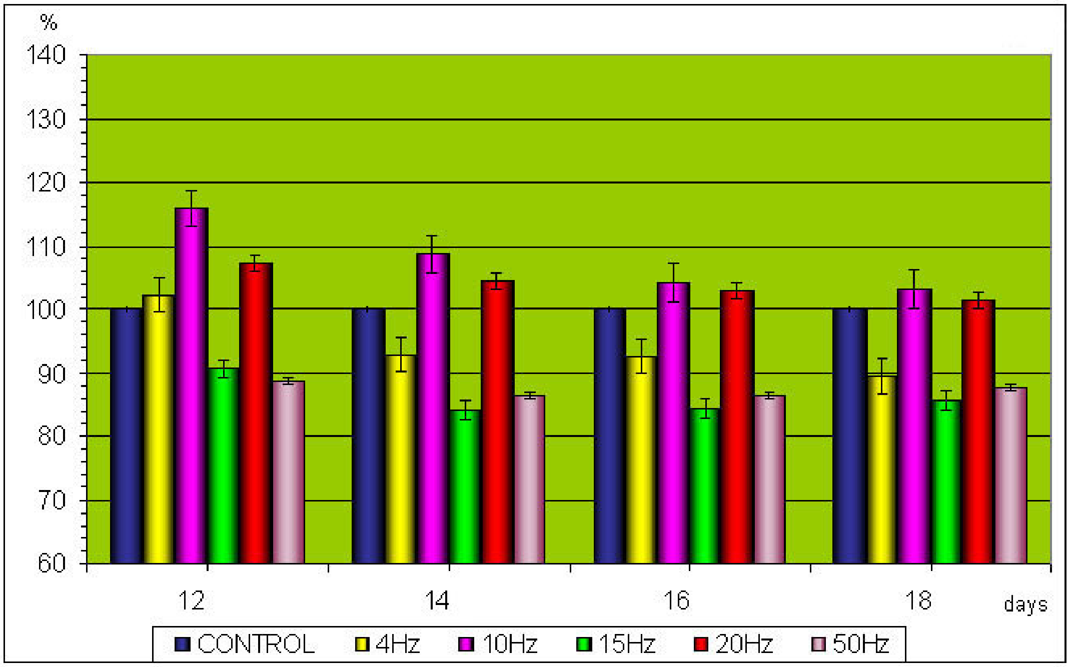

**FIGURE 10.** The time-dependent process of germination during 18-days incubation in nontreated (control) and ELFV-pretreated DW. On abscissa, the time (in days) of seed incubation; on ordinates, percent of the changes of germ length compared to the control.

## DISCUSSION

Water is the main component (60–90%) of the cell and it is in water that the cell metabolic process takes place. Hence, one can assume that an insignificant change in the physicochemical state of both extra- and intracellular water can substantially affect the cell's metabolic activity. It is known that the energy that is necessary for changing the cluster structure of water (it is this structure that chiefly determines the physiological properties of water) is lower than that needed for rupturing hydrogen bonds[1]. Therefore, one can also assume that water can be a universal target through which the extremely weak physical forces act[4,5].

The previous studies have shown that LF EMF and ELFV had a similar frequency-dependant depressing effect on SEC of water and water solutions[6,7]. On the basis of these data, it was suggested that such frequency dependence was determined by the vibration of water dipole molecules. However, the valence angle of water molecules, which could be modified by changing the coulomb-coulomb interaction between oxygen electrons with noncompensate spin, also could serve as the target for EMF effect[1,3].

So, the biological meaning of the above-mentioned two possible pathways, valence angle changes and mechanical rotation of dipoles through which the physicochemical properties of water on the EMF exposure can be modulated, is the subject for present investigation.

The preliminary study of the frequency-dependant effect of EMF and ELFV on the functional activity of *Escherichia coli* has shown that these two factors had the opposite effect in the growth of *E. coli*[7,14].

The present experiments have shown that for comparative study of the biological effects of EMF and ELFV, plant seed hydration and germination serve as a very convenient model because of the possibility to study their effects in metabolic inactive (cold) and active (warm) mediums.

On the basis of the obtained data on time-dependent changes of seed hydration in nontreated DW during 72-h incubation, two phases could be distinguished: (1) the slow gradual elevation (first 48-h incubation) and (2) the phase of sharp increase (48- to 72-h incubation) of seed hydration (Fig. 2A). The last phase, which was absent in cold DW and corresponded to the active (metabolic) germination phase, was very sensitive to preliminary treatment of bathing aqua solution by EMF (Fig. 3) and ELFV (Fig. 7).

Previously, it was shown that EMF and ELFV effects on SEC of DW had similar frequency windows, 4 and 20 Hz, however, in the case of seed hydration, we have different frequency "windows" at different phases of seed incubation, which can be explained by interaction of water and seed components. The latter could serve as a subject for special investigation.

The obtained data have shown that in control DW based on the rate of seed dry weight changes, the period of 72 h of seed incubation can be distinguished into three phases: (1) fast decrease, first 2 h of incubation; (2) fast increase, 2–24 h of incubation; and (3) slow increase in cold or decrease in warm DW (24–72 h of incubation). The dramatic changes of seed dry weight kinetics in LF EMF- and ELFV-treated DW, compared to the control and their frequency-dependent characters, allow us to suggest that these factors-induced changes of water properties in the result of valence angle changes and vibration of water molecules dipoles can determine EMF effect on seed hydration, solubility of water components, and water binding in seed.

Thus, the obtained data allow us to come to the following conclusions:

1. The metabolic-dependent seed hydration, exudation of seed dry weight, and water binding in seed can be modulated by DW preliminarily treated by LF EMF and ELFV.
2. ELFV-induced changes of water properties could serve as a messenger for realizing the biological effects of LF EMF on seed germination and different function of various cells and organisms. To finalize these conclusions, more detailed investigation on LF EMF and ELFV effects on physicochemical properties of water and water solutions is necessary, which is subject for our current investigation.
